# Development and In Vivo Evaluation of a Novel Vitamin D3 Oral Spray Delivery System

**DOI:** 10.3390/pharmaceutics16010025

**Published:** 2023-12-24

**Authors:** Xin Yan, Enhao Lu, Zhuo Song, Yuexing Wu, Xianyi Sha

**Affiliations:** 1 Key Laboratory of Smart Drug Delivery, Ministry of Education, School of Pharmacy, Fudan University, Shanghai 201203, China; 22211030034@m.fudan.edu.cn (X.Y.); ehlu19@fudan.edu.cn (E.L.); 2 Shanghai JiaLanHai NanoTechnology Group Co., Ltd., Shanghai 200335, China; szt@jialanhai.com (Z.S.); wyx@jialanhai.com (Y.W.); 3 The Institutes of Integrative Medicine, Fudan University, Shanghai 200040, China

**Keywords:** vitamin D3, spray, formulas, localized therapy

## Abstract

Developing drugs that are highly selective to host tissues but are the least toxic remains one of the most difficult challenges in cancer treatment. Recent studies have shown that tumor cells from a variety of sources can express vitamin D3 receptors and that the response to vitamin D3 and its analogs is prone to growth arrest and cell death. However, conventional vitamin D3 drug formulations lack dose control and cannot target specific cells or tissues. The aim of this study was to prepare vitamin D3 nanospray for inhalation delivery route. This study evaluated the physical properties of the formulation (particle size distribution and biological stability), the total number of sprays per bottle, the spray volume per spray, and the loading variance of the spray. The optimized vitamin D3 spray formula is easy to spray, has fewer drips, and has a fast drying time. It can be stored for 3 months at 37 ± 2 °C temperature, 75 ± 5% relative humidity, and away from light, and can maintain biological stability. This study showed that compared with traditional nasal sprays, the spray has a larger fan angle (82.1 degrees) and beam width (104.88 mm), more symmetrical spray on both sides of the spray column, a faster coverage of the administration site, and a wider range, which is suitable for inhalation delivery routes.

## 1. Introduction

Nanoemulsion is the dispersion of two kinds of immiscible solution, water in oil (O/W) and water in oil (W/O), and the use of suitable surfactants to stabilize. The average droplet diameter obtained is usually <500 nm [1]. Small droplets give it a clear or hazy appearance, which differs from the milky whiteness of coarse milk (micron-sized droplets involved in multiple light scattering). Nanoemulsions can be made into several dosage forms, such as liquid, emulsion, spray, gel, aerosol [2], and foam, and can be administered by the same different routes, such as topical, oral, intravenous, intranasal, pulmonary, and intraocular. In this paper, inhalation administration is performed in the form of aerosol. They have a higher solubilization capacity than simple micellar dispersions and a higher kinetic stability than coarse emulsions. Their long-term physical stability is a direct result of the small droplet size, which compromises traditional instabilities such as emulsification, precipitation, and coalescence. Usually, Browne’s motion is strong enough to counteract the kinetic instabilities caused by gravity or viscosity. When administered orally, the minuscule size of droplets in nanoemulsion and their capability to solubilize very hydrophobic drugs provides a pathway to drastically increase the rate of drug dissolution and subsequently expected systemic bioavailability [3].

Epidemiologic studies have shown that vitamin D3 deficiency and insufficiency are widespread worldwide [4,5]. These results have led to a significant investment in vitamin D3 research. To study the pleiotropic effects of vitamin D3, scientists typically use capsules or tablets as the route of vitamin D3 administration [6,7]. However, despite being commercially available, little is known about the efficacy of oral spray vitamin D3, primarily absorbed in the mouth’s buccal, sublingual, and palatal membranes rather than the gastrointestinal tract. Emerging evidence also suggests that oral spray vitamin D3 may provide an accelerated route of absorption compared to capsules and may be beneficial to patients with gastrointestinal malabsorption.

When the active ingredient reaches the buccal–buccal, sublingual, or intestinal mucosa, it must first pass through the mucus layer of the epithelium and then through the epithelium into the body [8]. The mucus layer is a highly complex mucus-like substance covering the epithelium, dramatically influencing the ingredient’s diffusion [8,9,10]. Second, to reduce the negative impact of the first-pass effect, buccal and sublingual routes are noticed by researchers that the gastrointestinal tract, the hepatic first-pass, enzymatic degradation, and some chemical instability can be avoided [11]. Nanoparticles may encourage mucous membrane absorption and improve the buccal and sublingual route rate, raising active ingredients’ absorption rates and bio-availability [12,13].

This study aimed to develop a spray formulation containing vitamin D3 for the inhalation delivery route. In this study, different parameters were selected for a series of evaluations of the preparation, including the physical properties (particle size distribution, biological stability), the total number of sprays per bottle, the number of sprays per bottle, the amount and difference of sprays, and the bioavailability in rabbits (AUC, Cmax, Tmax). The authors aimed to fill the research gap in vitamin D3 sprays by providing solutions for clinical applications through ex vivo and in vivo studies to create a valuable reference for healthcare professionals.

## 2. Materials and Methods

### 2.1. Materials

Vitamin D3 nanosprays (Natural D3, sublingual sprays, 1000 IU per spray shot, technically empowered by patented NANOSYNERGY^®^ (Shanghai JiaLanHai NanoTechnology Group Co., Ltd., Shanghai, China) applied nanotechnology) Formulas: vitamin D3 (Cholecalciferol), deionized water, vitamin E—TPGS, glycerin (sourced from palm oil), medium-chain triglycerides, citric acid, sodium citrate, rebaudioside A, natural tropical flavor, potassium sorbate. Vitamin D3 soft gelatin capsule (commercial Vitamin D capsule in the market, 400 IU per capsule, purchased from local pharmacy). Formulas: vitamin D3 (Cholecalciferol), purified water, glycerin, soy oil gelatin. The spray actuator used for this nanospray product is a pre-metered, non-aerosol spray actuator provided by NanoSynergy Worldwide, INC with its proprietary NanoMist^®^ (Shanghai JiaLanHai NanoTechnology Group Co., Ltd., Shanghai, China) dispensing technology. All other chemicals were of analytical grade.

### 2.2. Morphological and Physical Properties

The surface morphology of the samples was determined by transmission electron microscopy (TEM, JEM-1400 plus, Japan Electronic Corporation, Tokyo, Japan). The sample preparation process is as follows: take an appropriate amount of vitamin D3 spray liquid placed on the carbon support film copper mesh, wait for all the water to evaporate, then take an appropriate amount of 2% phosphotungstic acid to submerge the copper mesh for negative staining, wait for all the water to evaporate, with the help of a transmission electron microscope to observe the morphology and photograph.

### 2.3. Preparation of Vitamin D3 Nanospray

**Figure 1:**
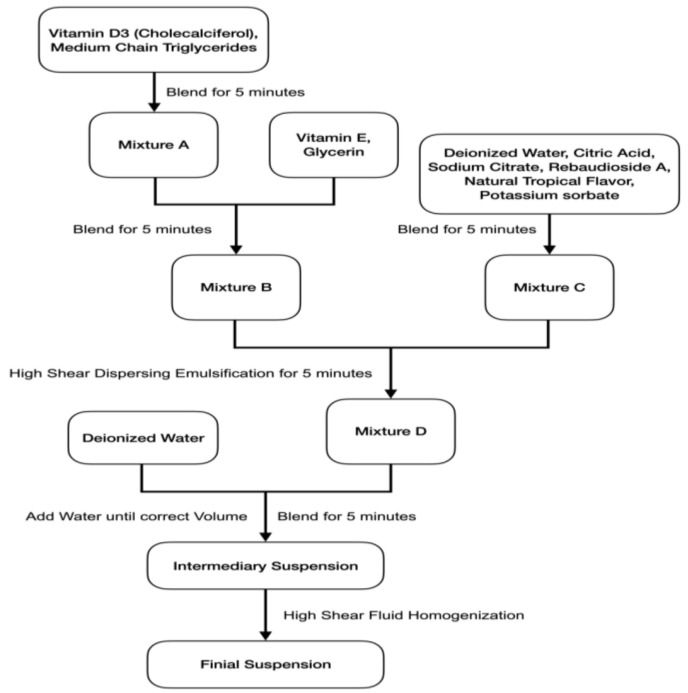
Process flow chart of vitamin D3 nanospray.

The preparation process of vitamin D3 nanospray is shown in Figure 1. A total of 100.0 mg of Cholecalciferol (4 million IU) was weighed with digital balance and placed into a 5 mL snap-lock microcentrifuge tube. Then, 3900 mg of medium-chain triglycerides was considered and added to the Cholecalciferol. A vortex mixer mixed Cholecalciferol and medium-chain triglycerides. Then, ultrasonic vibration was utilized to ensure a complete dissolution to obtain 4 g of 2.5% *w*/*w* (1 million IU/g) Cholecalciferol in medium-chain triglycerides (Mixture A).

We accurately weighed 0.50 g of 2.5% *w*/*w* Cholecalciferol in medium-chain triglycerides (contains 0.5 million IU/g) into a 50 mL Erlenmeyer Flask. We also accurately weighed pre-formulated and set amounts of glycerin and vitamin E, added to the 50 mL Erlenmeyer Flask. The total volume is approximately 20 mL. They are stirred for 5 min with a magnetic stirrer with the free rotor and ultrasonic vibration to ensure a complete dissolution to obtain Mixture B.

We accurately weighed the pre-formulated and set amount of citric acid, sodium citrate, rebaudioside A, natural tropical flavor, potassium sorbate, and deionized water. The total volume is approximately 30 mL, placed into a 100 mL Erlenmeyer Flask. They are stirred for 5 min with a magnetic stirrer with the free rotor and ultrasonic vibration to ensure a complete dissolution to obtain Mixture C.

We gradually added Mixture B into Mixture C while stirring and rinsed the 50 mL Erlenmeyer Flash 3 times with deionized water to ensure Mixture B was transferred completely. We continuously stirred for 5 min and then performed ultrasonic vibration to generate the emulsification of Mixture D.

The total volume of mixture D was adjusted by pipetting deionized water until the exact volume was 70 mL, and 500,000 IU cholecalciferol per 70 mL (1000 IU cholecalciferol /0.14 mL) was obtained through the intermediate suspension.The intermediary suspension was processed with high-shear fluid homogenization by technology provided by Microfluidics™ IDEX MPT Group, Westwood, MA 02090, USA, to obtain the final break.

This final suspension is 1000 IU Cholecalciferol/0.14 mL theoretically, and with the spray mentioned above actuators mentioned above, each complete press of spray mist (0.140 mL) will dispense 1000 IU of Cholecalciferol.

### 2.4. Determination of Liquid Particle Size and Particle Size Distribution of Sprays

The particle size and distribution of vitamin D3 nanospray-filled liquids at different dilutions were determined by dynamic light scattering using a Malvern particle size analyzer. Measurement conditions were as follows: measurement temperature is 25 °C, equilibrium time is 120 s, scattering angle is 90°, and dilution solution is sterile water. Before the first experiment, the background was tested under dark conditions for calibration. To perform the test, the sample’s nebulizer cup was positioned so that the laser beam passed through the center of the spray area.

### 2.5. Determination of Droplet Size and Its Distribution in Vitamin D3 Nanospray

Three samples of the spray were taken, and each model was sprayed with five sprays before testing, after which the samples were placed vertically on the automatic sprayer, and the nozzle was fixed so that the laser beam passed through the center of the spray range.

According to the above measurement conditions, we determined the particle size distribution at a distance of 3 cm (the vertical distance between the tip of the nozzle and the center of the laser beam) and reported the particle sizes Dv10, Dv50, and Dv90, respectively (the droplet sizes corresponding to a cumulative volume fraction of 10%, 50%, and 90%, respectively).

### 2.6. Determination of the Total Number of Sprays per Bottle of Nanospray

Take four bottles of test products, remove the cap, shake thoroughly, follow the instructions, release the contents into the collection container, and press the spray pump (pay attention to each spray interval of 5 s and slowly shake) until the spray is exhausted, and calculate the spray times, respectively.

### 2.7. Determination of the Amount per Spray of Nanosprays

According to the instructions, take one bottle of the test product, discard several spraying times, wipe, precision weighing, spray once, wipe, and then precision weighing. The difference between the two weights before and after is one spray volume. A total of 10 spray volumes were measured before the marked spray (initial three spray volumes), in the middle (n/2 spray up four spray volumes, n is the considerable total spray volume), and after (the last three spray volumes). The average of the above ten spray volumes is calculated. The test is repeated for three more bottles.

### 2.8. Loading and Loading Difference

Take 20 bottles of the test product, according to the method specified under each variety, to find the filling amount of each content and the average filling amount.

### 2.9. Evaluation of the Spray Mode of Vitamin D3 Nanospray

The Spray VIEW laser image system was used for the determination. Before each test, the spray agent should be sprayed five times before triggering. The test should be carried out at 3 cm (the distance from the nozzle to the laser beam). The instrument parameters were set as follows: camera position at 10.0 cm; camera height of 21.0 cm; lens aperture of 1.2; laser position at 6.0 cm. High-speed cameras were used to take spray longitudinal cross-section photographs, which are spray geometry photographs. The spray angle and maximum spray width were reported.

### 2.10. Biostability Assessment

To evaluate the stability of this nanospray, accelerated testing was performed by a third-party CMA47 laboratory of Beijing Hontest Technology Development Co., Ltd. (Beijing, China), in 2021. Three batches, 180 bottles each batch, a total of 540 bottles of vitamin D3 nanospray were stored under conditions, temperature 37 ± 2 °C, relative humidity (RH) 75 ± 5%, out of direct sunlight exposure, for three months, and amounts of active ingredients, heavy metals, microbiological contamination, TDS and pH were analyzed initially (at 0 months), after 1, 2, and 3 months.

### 2.11. In Vivo Pharmacokinetic Studies

#### 2.11.1. Animal Experimentation

Twelve New Zealand White rabbits, weighing 2.5 ± 0.2 kg, half male and half female, were randomly divided into two groups of 6 rabbits (3 females + 3 males) for pharmacokinetic study. In the oral preparation group, each rabbit was given 1.2 mL of vitamin D3 soft capsule content, and in the oral spray group, each rabbit was given two sprays of vitamin D3 oral spray. Blood was collected from the marginal vein of the ear of rabbits at 17-time points, before and 0.5, 1, 2, 4, 6, 8, 12, 24, 36, 48, 72, 96, 144, 192, 240, and 288 h after the administration of vitamin D3, in 2 mL EP tubes, and was collected in pre-filled with sodium heparin. The blood was collected from the rabbit ear marginal vein and collected in a 2 mL EP tube pre-filled with sodium heparin, centrifuged at 10,000× *g* for 10 min at 4 °C, and the plasma samples were put into brown 1.5 mL EP tubes and stored in an ultra-low temperature refrigerator at −80 °C, waiting for the subsequent processing. Rabbits were fasted for 12 h before administration, blood sampling was performed in a clean-grade barrier area of the animal house, and any other medication was prohibited for two weeks before the test and until the end of the entire examination.

#### 2.11.2. Sample Collection and Handling

Remove the plasma sample from the refrigerator at −80 °C, melt at room temperature, and vortex for 30 s; precisely aspirate 500 μL of plasma, put it into a 15 mL brown centrifuge tube, add about 1 mL of methanol to precipitate proteins, exactly add 50 μL of the internal standard working solution (25-OH-VitD3-d6 methanol solution at 500 ng/mL), add about 5 mL of n-hexane and vortex for 1.5 min, centrifuge for 5 min at 1000 g and absorb the upper layer of n-hexane phase about 4 mL, blow to dryness at 40 °C under nitrogen pressure. Centrifuge at 1000× *g* for 5 min at 4 °C, take up about 4 mL of the upper hexane phase, and blow to dryness at 40 °C with nitrogen. After blow-drying, store the EP tubes in the refrigerator at −20 °C for subsequent measurement. Then, 12 h before the measurement, take out the above samples from the fridge at −20 °C, add 100 μL of methanol to dissolve, vortex for 1 min, centrifuge at 10,000× *g* for 5 min at 4 °C, and then take 80 μL of supernatant into an auto sampling vial, and then inject 10 µL of the supernatant into the vial to measure the concentration of the drug and calculate the attention of the drug by the internal standard method.

#### 2.11.3. Chromatographic Conditions and TEM

Waters C18 stationary phase Xbridge 250 × 4.6 mm with a particle size of 5 μm was used for gradient elution with 0.1% formic acid–water phase and 0.1% formic acid–methanol phase, and 50 µL volume was injected into the chromatographic system. The column temperature was set at 40 °C and the flow rate was 0.4 mL/min. Then, the sample was injected into a high-performance liquid chromatograph (Waters, Milford, MA, USA) equipped with a PDA detector (Waters, Milford, MA, USA). Empower 3 software (version WKB193113) was used to determine the sample concentration using linear equations. The surface morphology of the samples was determined by transmission electron microscopy (TEM, JEM-1400 plus, Japan Electronic Corporation). The sample preparation process is as follows: take an appropriate amount of vitamin D3 spray liquid placed on the carbon support film copper mesh, wait for all the water to evaporate, then take an appropriate amount of 2% phosphotungstic acid to submerge the copper mesh for negative staining, wait for all the water to evaporate, with the help of a transmission electron microscope to observe the morphology and photograph.

#### 2.11.4. Pharmacokinetic Parameters

AUC_0~t_ is calculated by the trapezoidal method based on the measured blood concentration at each time point, AUC_0~∞_ accrues the area of the extrapolation of the time-of-drug curve based on AUC_0~t_, Cmax is the maximum concentration reached, Tmax is the time to get the utmost attention, t_1/2_ refers to the half-life, and Ke refers to the elimination rate constant.

## 3. Results

### 3.1. Appearance, Particle Size, Size Distribution, and Particle Morphology

**Figure 2:**
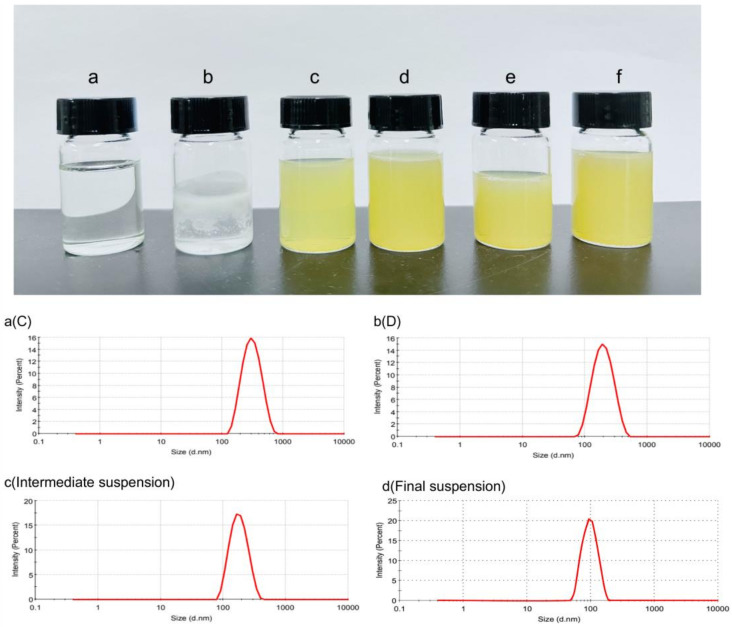
Appearance and particle size characterization of the solution during preparation: a, b, c, d, e, and f represent the appearance morphology of solution A, B, C, D, intermediate suspension, and final suspension, respectively; (**a**) (C) is solution C, (**b**) (D) is solution D, (**c**) (Intermediate suspension) is the intermediate suspension, and (**d**) (Final suspension) is the final suspension.

As shown in Table 1, the average particle size of vitamin D3 nanospray contents was about 280 nm, and the average particle size was about 160 nm after different dilution multiples, with small particle size changes and narrow particle size distribution (PDI was all less than 0.15). PDI is a measure of sample heterogeneity based on size, and values of 0.2 and below are generally considered acceptable in polymer-based nanoparticle materials. This relatively low PDI (0.008) indicates that the analyzed sample is reasonably uniform in particle size. The appearance and particle size characterization of the solution in the preparation process are shown in Figure 2.

**Figure 3:**
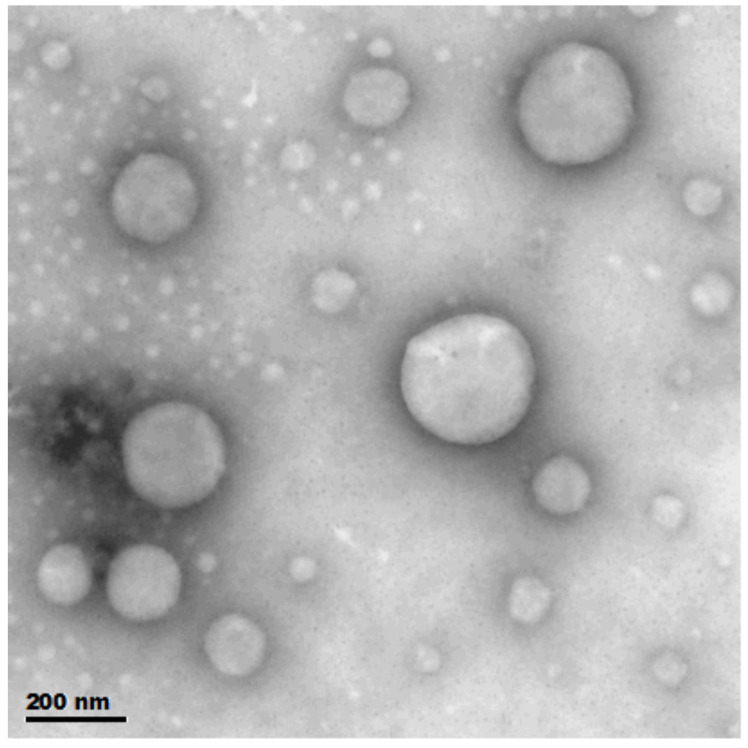
Morphology of the sample observed under transmission electron microscopy. Scale bar = 0.2 µm.

In addition, we characterized the sample morphology using transmission electron microscopy (TEM). Transmission electron microscopy results showed that the vitamin D3 nanosprays were all circular and spherical with a uniform surface and uniform size, and the characterization results are shown in Figure 3.

### 3.2. Characterization of Nanospray

#### 3.2.1. Aerosol Droplet Size and Distribution

**Figure 4:**
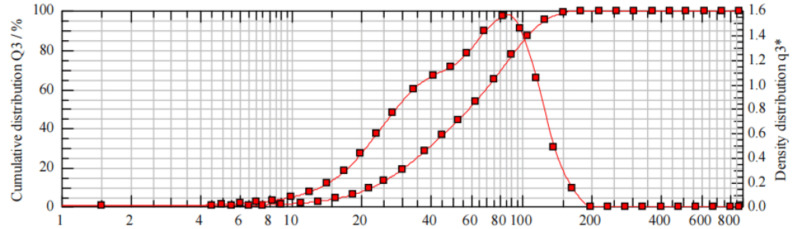
Droplet size and distribution of vitamin D3 nanospray at 3 cm. * *p* < 0.05.

As shown in Table 2 and Figure 4, the geometric mean particle size (VMD) of the droplet at a detection distance of 3 cm is 61.63 ± 1.72 μm.

#### 3.2.2. Nanospray Total Spray Times Per Bottle

The results showed that the average number of sprays of the vitamin D3 nanospray was 188, the total number of sprays of the vitamin D3 nanospray was all higher than the labeled full spray (180), and the average number of sprays of vitamin D3 nano-particle spray was 177 when the dose reached 80% to 120% of the labeled amount (*n* = 4).

#### 3.2.3. Nanospray Volume Per Spray

The vitamin D3 nanospray averaged 0.1347 g to 0.1476 g per spray. The vitamin D3 nanoparticle spray had a dose per spray range of 96.22% to 105.66% of the labeled amount and met 80% to 120% quality requirements. The vitamin D3 nanospray was used in various applications (*n* = 4).

#### 3.2.4. Volume and Volume Differences

The vitamin D3 nanospray loading and loading variance showed that the average loading of the vitamin D3 nanospray was 26.7 mL (*n* = 20). No samples were found to exceed the loading variance limit when the loading of each one was compared to the average loading, which complied with the quality requirement of the loading variance of the sprays (±7.5%).

#### 3.2.5. Spray Mode of Vitamin D3 Nanospray

**Figure 5:**
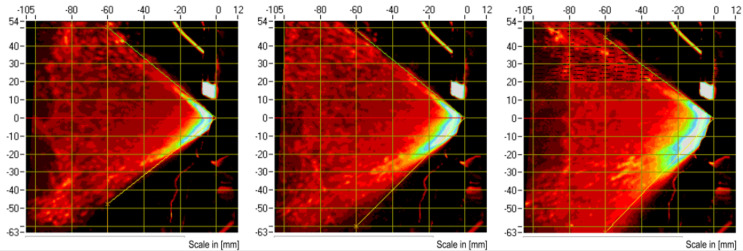
Vitamin D3 nanospray pattern at 3 cm (geometry).

As shown in Figure 5, we used the Spray VIEW laser imaging system to measure the vitamin D3 nanospray. The spray produced an average fan angle of 82.10 degrees and an average wisp width of 104.88 mm (*n* = 3). It can be seen that the angle and width values are relatively large, and both sides of the spray column are more symmetrical, which is directly related to the type of pump used in the formulation. The video screen of the spray is shown in the Supporting Information.

### 3.3. Biological Stability

As shown in Table 3, we practically determined the active ingredients, microbial contamination, total dissolved solids, and pH of vitamin D3 nanospray by accelerated experiments. The results showed that the stability of vitamin D3 was high under the condition of shading, 37 ± 2 °C and 75 ± 5% relative humidity, and the stability decreased slightly with the extension of storage time, but the effect was not significant. Secondly, the results of microbial contamination showed that the total plate counts of microorganisms ranged from 50 to 95 CFU/mL with the extension of storage time, of which the concentration of *E. coli* was always less than 0.3 MPN/mL, and the concentration of yeasts and molds was always less than 10 CFU/mL. In addition, the percentage of total dissolved solids ranged from 32.0% to 32.4%.

### 3.4. In Vivo Pharmacokinetic Studies

**Figure 6:**
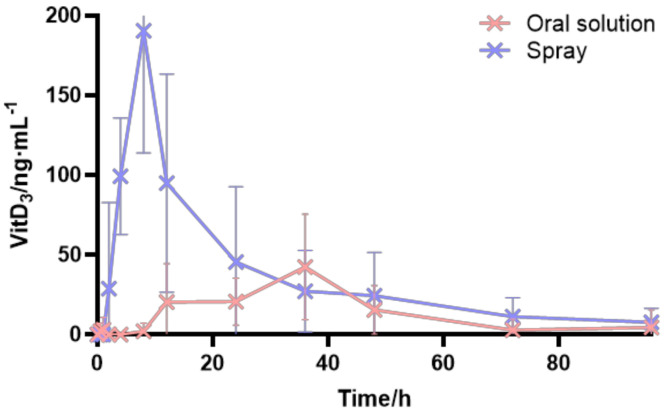
Plasma mean of VitD3 concentration-time curves in rabbits in the oral administration group vs. oral spray administration group.

**Figure 7:**
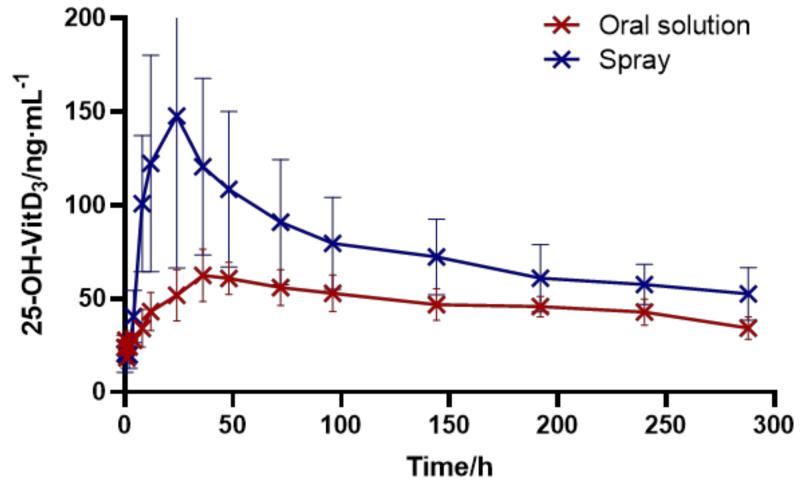
Plasma mean of 25-OH-VitD3 concentration-time curves of rabbits in the orally administered and orally sprayed distributed groups.

In this study, the drug was administered by intragastric administration and oral spray, respectively. In the intragastric administration group, 1.2 mL VitD3 soft gel contents were intragastric administration for each animal, and each animal in the oral spray group was given two doses of vitamin d3 spray. The average blood concentration of vitamin d3 in rabbits in the two groups is shown in Figure 6 and Figure 7, and the pharmacokinetic parameters are shown in Table 4. In addition, 25-OH-Vit D3 is the main form of Vit D3 in the somatic circulation, and it is also the main target product for bioavailability determination, so the pharmacokinetic parameters were calculated for 25-OH-Vit D3 only. As shown in Table 5, after oral spray administration (1000 IU of VitD3 per spray), the blood concentration of 25-OH-VitD3 peaked rapidly. Then, it declined, with a Tmax of 26 h. In contrast, after oral administration (each capsule containing 250 mg of the contents of the oily liquid, equivalent to 400 IU of VitD3), the Tmax was 40 h, and the blood concentration of 25-OH-VitD3 in the oral spray group was consistently more remarkable than that in the said group (*p* < 0.01). There was a significant difference in Cmax between the two modes of administration, with a Cmax of 55.88 ng/mL for oral administration and 139.93 ng/mL for oral spray administration (*p* < 0.01). The elimination rate constant Ke was 0.0041 for oral administration and 0.0037 for oral spray administration, indicating that the drug would be eliminated faster in the body after oral administration. Compared with the traditional gastrointestinal oral route of administration, the bioavailability of 25-OH-VitD3 in rabbits after Vit D3 oral spray administration was about 1.7 times higher than that of gavage administration, suggesting that Vit D3 oral spray has the dosage form advantage of rapid drug absorption and high bioavailability.

## 4. Discussion

Several factors influence the absorption of vitamin D3, including gastric, pancreatic, biliary, and various organ metabolisms [14]. The serum concentration of 25-hydroxyvitamin D is a good indicator of long-term vitamin D levels in the body. However, it is insensitive to a single dose of vitamin D and does not exceed the normal range unless taken over a long period. Currently, malabsorption of vitamin D is widespread because vitamin D is a relatively non-polar sterol, which must be solubilized by admixture with bile salt micellar solutions to be absorbed in the aqueous phase [15]. If normal pancreatic or biliary secretion is interfered with, this process is severely inhibited, leading to a deficiency of the appropriate vitamin D levels in the body [16,17]. The first-pass effect is a phenomenon in which a significant reduction in active ingredients’ concentrations due to the components being metabolized by specific body organs before elements reach the systemic circulation [18]. Most of this effect often occurs in the liver and the lungs, vasculature, gastrointestinal tract, and other tissues in the body. Considering the problems of malabsorption in intestinal disorders as well as possible malabsorption and complexity of oral formulations, we developed a novel oral nanospray of vitamin D3, in which vitamin D3 is suspended in the aqueous phase and readily enters the corpuscular circulation via the sublingual mucosal route. VitD3 itself is not biologically active and is rapidly converted into the less biologically active 25-OH-VITD3 in the liver and then further converted into the biologically active but less stable 1α,25-(OH)2-VitD3 in the kidney to perform biological functions. Among them, 25-OH-VitD3 is the main form of VitD3 in somatic circulation and the primary target substance for pharmacokinetic assays. Oral exogenous VitD3 is absorbed in the GI tract and converted to 25-OH-VitD3 by the hepatic first-pass effect, which is present in plasma. In contrast, VitD3 oral sprays may have a mucosal absorption pathway and may be present in plasma as a prototype after administration. In a healthy rabbit model, vitamin D3 soft gels increased the plasma levels of 25-OH-VitD3 from 19.08 ng/mL to 25.52 ng/mL, whereas oral spray increased the plasma levels of 25-OH-VitD3 from 15.00 ng/mL to 44.02 ng/mL.

Our findings suggest that oral spray vitamin D3 is as effective as capsule supplements in increasing plasma 25-OH-VitD3 concentrations. However, we hypothesize that the ability of vitamin D3 oral sprays to bypass the intestinal absorption pathway in the clinic is likely to prove more advantageous in individuals with gastrointestinal malabsorption syndrome and dysphagia (e.g., the elderly, young children, and infants) because regardless of the absorption pathway, the oral spray and capsule-formulated vitamin D3 must first undergo hepatic hydroxylation before the formation of 25-(OH)-VitD3. Thus, for older people and infant populations, the long-term benefits of oral sprays are attributed to their ability to enhance the degree of vitamin D3 absorption in the body rather than how quickly it enters the body’s circulation. Future research in this area should focus on comparing the effectiveness of oral spray vitamin D3 supplementation with other methods in patients with gastrointestinal malabsorption. If our findings are replicated, or oral spray vitamin D3 is superior to capsules in these individuals, oral spray supplementation could provide a non-invasive alternative to injections, thereby reducing the management burden on patients.

## 5. Conclusions

In this study, a novel vitamin D3 nanospray was prepared, and its formulation was evaluated for physical properties (particle size distribution and biostability), total number of sprays per vial, spray volume per spray, and spray loading and loading variability. The optimized vitamin D3 spray formula is easy to spray, has fewer drips, and has a fast drying time. It can be stored for 3 months at 37 ± 2 °C temperature, 75 ± 5% relative humidity, and away from light, and can maintain biological stability. This study showed that compared with traditional nasal sprays, the spray has a larger fan Angle (82.1 degrees) and beam width (104.88 mm), more symmetrical spray on both sides of the spray column, faster coverage of the administration site, and a wider range, which is suitable for inhalation delivery routes. In addition, an in vivo pharmacokinetic study showed that compared to the oral route of administration, the peak concentration Cmax of the major active metabolite of vitamin D3, 25-OH-VitD3, after oral spray administration was significant. The peak concentration of 25-OH-VitD3 after oral spray administration was significantly higher than that after gavage administration (*p* < 0.01), and the peak time Tmax was also substantially faster than that after gavage administration (*p* < 0.01), suggesting that the oral spray of Vit D3 has the advantages of rapid drug absorption and high bioavailability.

## Figures and Tables

**Table 1:** Particle size and particle size distribution of vitamin D3 nanospray contents (*n* = 3).

Samples	Average Size (nm)	PDI	D10 (nm)	D50 (nm)	D90 (nm)
stock solution	287.1	0.086	192	302	480
Diluted 5 times	180.0	0.100	120	194	317
Diluted 10 times	168.8	0.086	116	176	271
Diluted 20 times	163.7	0.092	112	172	267
Diluted 50 times	160.0	0.095	113	168	252

**Table 2:** Measurement results of droplet size and distribution of vitamin D3 nanospray (*n* = 3). * *p* < 0.05, ** *p* < 0.01, *** *p* < 0.001.

Samples	Particle Size (μm)			
	Dv10	Dv50	Dv90	VMD
1	21.81	54.32	106.24	60.07
2	22.51	55.98	108.29	61.35
3	22.48	58.61	112.34	63.47
Average Value	22.27 ***	56.30 *	108.96 **	61.63
SD	0.40	2.16	3.10	1.72

**Table 3:** Determination of Cholecalciferol, PH, and total dissolved solids (TDSs) in accelerated testing (*n* = 3).

Parameters	Initial (0 Month)	After 1 Month	After 2 Months	After 3 Months
Average active ingredient content (μg/bottle)	381	362	322	295
PH	4.1	4.1	4.1	4.1
Average total Dissolved Solids (TDS)	32.1%	32.3%	32.1%	32.2%

**Table 4:** Main in vivo pharmacokinetic parameters in rabbits (*n* = 6). * *p* < 0.05, ** *p* < 0.01.

Sample		T_max_	C_max_	k_e_	t_1/2_	AUC_0-t_	AUC_0-∞_
		h	Ng/mL	h^−1^	h	ng·h/mL	ng·h/mL
orally administered groups	Mean	40.00	55.88	0.0041	219.44	11,167.25	19,701.30
	SD	6.20	10.86	0.0025	119.40	1333.13	5178.06
spray administration group	Mean	26.00 **	139.93 **	0.0037 *	221.68 *	19,766.80 **	33,358.23 **
	SD	4.90	82.00	0.0016	108.50	6761.10	0.5

**Table 5:** Relative bioavailability of 25-OH-VitD3 in rabbits of two oral formulations of Vit D3 (*n* = 6).

Parameters	Unit	Gavage (mL)	Oral Spray (g)	Relative Bioavailability (Oral/Subcutaneous)
AUC_0-t_	ng/mL·h	11,167.25 ± 1333.13	19,766.80 ± 6761.10	177.01%
AUC_0-∞_	ng/mL·h	19,701.30 ± 5178.06	33,358.23 ± 12,233.5	169.32%

## Data Availability

The data presented in this study are available upon request from the corresponding author.

## Supplementary Materials

The following supporting information can be downloaded at: https://www.mdpi.com/article/10.3390/pharmaceutics16010025/s1, The video screen of the vitamin D3 nanospray.
